# Living With a New Normal: Self‐Identities of Women With Breast Cancer in Nigeria

**DOI:** 10.1002/cnr2.2148

**Published:** 2024-09-22

**Authors:** Aisha Abimbola Adaranijo, Jimoh Amzat, Dejo Abdulrahman, Kehinde Kazeem Kanmodi

**Affiliations:** ^1^ Department of Sociology Federal University Lokoja Lokoja Nigeria; ^2^ Department of Sociology Usmanu Danfodiyo University Sokoto Nigeria; ^3^ Department of Sociology University of Johannesburg Johannesburg South Africa; ^4^ School of Health and Life Sciences Teesside University Middlesbrough UK; ^5^ Faculty of Dentistry University of Puthisastra Phnom Penh Cambodia; ^6^ School of Dentistry University of Rwanda Kigali Rwanda; ^7^ Cephas Health Research Initiative Inc Ibadan Nigeria

**Keywords:** breast cancer, new normal, Nigeria, qualitative research, self‐identities

## Abstract

**Background:**

Breast cancer is the most prevalent cancer for women in Nigeria, representing 25% of all cancers in women. How do women self‐identify with the new realities of living with breast cancer before, during and after treatment?

**Aims:**

This study aims to examine the self‐identities of 22 women with breast cancer in Nigeria.

**Methods:**

The paper relies on grounded theory research method to collect data, analyse and capture the processes of self‐identity formation.

**Results:**

The qualitative data analysis reveals the basic social process within symbolic interactionism that describes how breast cancer survivors perceive their agency and how new self‐identities emerged from the new normal of living with breast cancer. A framework of three self‐identities emerged from the data: (1) valued self‐identity before breast cancer, (2) dependent and determined self‐identities during treatment and (3) devalued self‐identity post‐treatment.

**Conclusion:**

This study should help caregivers understand the profound perpetual psycho‐emotional impact that breast cancer has on sufferers and survivors.

## 1 Introduction

In Nigeria, breast cancer stands out as the most prevalent cancer among women, accounting for 25% of all cancers [1]. Globally, two significant social variables associated with breast cancer are age and race. Age positively correlates with the incidence of breast cancer, with most women receiving a diagnosis after the age of 40 [2]. The prevalence of breast cancer among younger women varies across races. While breast cancer is more common in Caucasian women than in Africans overall, in women under 35, breast cancer is more than twice as prevalent in African women [3, 4, 5]. Like their African American counterparts, women living with breast cancer in Nigeria experience breast cancer earlier than Caucasians [6, 7]. Some factors and processes highlight the experience of women with breast cancer in Nigeria, which may significantly differ from other documented cases. Young women, usually between 35 and 40, of African origins, often report a late stage of cancer [5, 8], more cases are detected at later stages, and access to appropriate treatment is limited compared to other contexts represented in the literature.

Studies have reported that women with breast cancer have divergent survivorship views and differ in how they accept or reject the survivorship identity [9, 10]. To thoroughly comprehend the experience of breast cancer for an Indigenous Black woman, this study relied on a grounded theory research method for data collection and analysis and to develop typologies explaining the pathways to a new self‐identity before, during and after treatment, and the concept of the ‘new normal’ of life living with breast cancer. In most cases, breast cancer diagnosis often brings profound physical, emotional and social changes, compelling women to navigate a ‘new normal’. Also, the concept of self‐identity becomes central as women with breast cancer adapt to life post‐diagnosis, balancing personal, familial and societal roles.

Self‐identity refers to the perception or recognition of one's characteristics, qualities and beliefs that make up one's concrete or perceptual self‐image. It is how individuals understand and define themselves in relation to the world around them including the consideration of their peculiar condition. Self‐identity is dynamic and can change over time due to personal experiences, social interactions and life transitions. Women with breast cancer frequently face significant changes concerning their self‐identity. The physical transformations due to surgery, chemotherapy and radiation, such as mastectomy or hair loss, can alter their body image, sense of femininity and overall identity. In Nigeria, societal expectations regarding femininity and motherhood add layers of pressure, often exacerbating the emotional and psychological burden of the disease. Hence, this study examines this ‘new normal’ following breast cancer diagnosis and self‐identities of women with breast cancer in Nigeria.

From a sociological perspective, this article (with data from a more comprehensive study) using grounded theory approach (GTA) examines the illness identity of women who have undergone treatments for breast cancer in a Nigerian state. Grounded theory is from the interpretive paradigm, which explores social processes and actions in human behaviour. The grounded theory method emphasises developing a theory or framework that explains social phenomena through constant comparative analysis, including context issues. For instance, unlike in the Global North, treatment is not adequately accessible and affordable in Nigeria. With no national or subnational governmental policies and programmes on early detection and treatment, cancer treatment is usually out‐of‐pocket. There is also no structured insurance scheme for cancer treatment. The National Health Insurance Scheme (NHIS), which covers less than 5% of Nigeria's population of over 206 million [11], does not cover cancer treatments. An overview of the breast cancer situation reveals that only 14 cancer treatment centres are available in Nigeria, in 10 states (out of 36), mainly in southern Nigeria, with only four providing radiation therapy. Due to certain socio‐cultural and economic factors that delay access to proper care, the majority of women receive a diagnosis at the late stage (metastasis) of the disease. Therefore, the experience of breast cancer for young African women in Nigeria is different from that of an African American young woman with breast cancer.

Interest in people with chronic illnesses has generated various models to examine and understand the indicators and factors involved in health, illness and disease. Interpretive sociology developed a view of people as social agents. The ill persons became the main interest, with the patient's lived narratives being the prime material in the research. The subjectivity of the illness becomes the focus in terms of, for instance, the disruption the illness brings, adaptation and coping strategies/styles as well as the value of self, discovering self, the deconstruction and reconstruction of self and so on. However, regardless of the basis of the methodology used, a sociological approach primarily attempts to look beyond the factors associated merely with an understanding of a disease. A sociological approach would be inclined to study a patient as a ‘whole person and as such, illness could only be fully understood by taking account of the wider social and cultural context in which physical and mental conditions are observed, diagnosed and treated’ [12].

The body of literature concerning the experiences of individuals living with breast cancer worldwide is extensive [13, 14, 15, 16]. This literature examines the diverse experiences and processes within the socio‐cultural contexts of the research, with a particular focus on the notion of women living with the disease as survivors. Survivorship is understood as an ongoing process involving negotiation and adaptation to the physical, psychological and social challenges associated with breast cancer. Charmaz's grounded theory works [17, 18, 19] offer valuable insights into how survivorship is influenced by socio‐cultural contexts, which profoundly shape one's identity and sense of self. In the specific context of the Global South, and Africa in particular, there arises the question of the transferability of knowledge from existing literature, considering the multitude of factors and socio‐cultural contexts that intersect in the experience of breast cancer for women.

## 2 Methods

A constructivist GTA was selected to guide the understanding of breast cancer experience in the Nigerian context. The choice for using GTA was because the comprehensive study was exploratory and sought to provide answers as to what exists concerning the lived experience and the social construction of the self while living with breast cancer in the Global South. Hence, the choice of qualitative methods and GTA (in particular) is also because of the sensitive nature of the subject—the general emotional reaction associated with breast cancer—and because the issue is deeply rooted within the knowledge and understanding of the sufferers [20, 21, 22, 23]. The GTA, being an inductive process, allows varied data collection and analysis to explain illness behaviour of those experiencing breast cancer, with the development of a framework about self‐identities concerning a new normal of living with breast cancer. The study was approved by the Institutional Review Board of the Federal Medical Centre Lokoja, Kogi State, Nigeria.

The study initially purposively selected 10 respondents. Through theoretical sampling, more respondents were selected for interview. Cumulatively, 30 women were recruited. During the study, eight women withdrew consent. Following the grounded theory process [22], data analysis started as soon as transcription commenced and continued until data saturation was achieved (Adaranijo et al. 2022) [24, 25]. During this process, the data were coded and categorised. Memos/field notes were also used for comparisons in the process until data saturation was achieved. The fundamental process for analysis with grounded theory was ensured, including openness throughout the study process.

Theoretical sampling is very vital in grounded theory methodology; it must be carried out during the sampling and data collection to facilitate the development of the model or theory. This was applied by carefully reinterviewing some participants and modifying questions to clarify uncertainties that arose from the data, test the interpretations observed and build the emerging theory until theoretical saturation was reached. Six of the participants were reinterviewed based on the preliminary categories that emerged, subsequently, three new participants that were not part of the initially interviewed cohort were interviewed to analyse the gaps raised. In total, 14 participants were interviewed twice and nine participants were interviewed thrice in an iterative, systematic process of data collection guided by ongoing analysis of the data.

The study population comprised 22 females aged 26–61, with a median age of 38 and at various stages of cancer from Stages 1–4 (Table 1). Participants included both inpatients and outpatients—women living with breast cancer—and the stretch of varied illness narratives. There were no inclusion or exclusion criteria because of the peculiarities of getting people with the disease to participate in research in Nigeria. The study sampled those available and willing to participate in the study in the selected treatment centre in an urban setting. For instance, most educated younger women with awareness of breast cancer live in urban areas. On the other hand, most older women with breast cancer usually with little or no formal education live in rural areas with low SES. The aforementioned context explains why the sample is relatively young.

**TABLE 1:** Sample characteristics (*n* = 22).

Characteristics	Categories	Number of participants
Age	25–35	5
	36–45	9
	46–55	4
	56–65	3
Education	No formal education	1
	Primary	6
	Secondary	13
	Post‐secondary	2
Occupation	Civil servant	4
	Small scale enterprise	6
	Professional	7
	Homemaker	5
Relationship status	Single	3
	Married	12
	Divorced/separated	4
	Widowed	3
Time since diagnosis	Up to 12 months	4
	1–2 years	7
	2–4 years	10
	More than 5 years	1
Stage of breast cancer	Stage 1	1
	Stage 2	8
	Stage 3	10
	Stage 4	3
Treatment	Surgery: mastectomy (+reconstruction)	21
	Other treatment	1
	Radiotherapy and/or chemotherapy	22
Location of interview	Participant's place of choice	15
	Place of worship	5
	Hospital	2

As with the interpretive approach, several limitations are worth highlighting. The choice of constructivist GTA was predicated on the assumption that it is an analytical research methodology. To mitigate bias, and ensure that the findings are not misconstrued to be based on the researchers' perspectives and positionality, reflexive journals were kept throughout the process of the research. Other sources, like the case files and interactions with the oncology teams, were also employed to collaborate on interpretations given by the women. Achieving theoretical saturation was challenging, as it was a subjective determination by the researchers. The data collection and analysis were time‐consuming and labour‐intensive, it was also heavily dependent on the researchers' skills in coding and memo writing. There were several meetings to agree on the themes, codes and categories that were emerging. As with the interpretive approach, the findings are context‐specific and may have limited generalizability to other populations or settings.

The Federal Medical Centre, Lokoja, was the recruitment site. The recruiting of participants was facilitated by the four general surgeons and the matrons‐in‐charge of both the outpatient and inpatient clinics in the hospital. Twenty‐two women aged between 25 and 64 were recruited for the study; 21 of these participants were diagnosed with late metastasis, mostly Stage 3 breast cancer.

Data were collected by conducting in‐depth interviews, memos and field notes. The interview guide was unstructured to discover new ideas as they emerged and further probes to ensure clarity, especially for theory development. The guide was modified as the data collection proceeded to further refine questions to elicit adequate information and further reflect the categories and concepts that needed more refinement [26]. In the interviews, questions were asked such as: When did you notice/understand the changes that were happening to your breast? Did you consult or discuss these changes with anyone? If yes, with whom did you discuss the changes? What advice did they give you? What did you do? When and how did you start seeking help for the problem? How did you get your diagnosis? When did you begin modern medical treatment? What do you think caused your breast cancer? What is your experience with breast cancer? What was your experience with the treatment? In your opinion, how can it be treated? How did you think of yourself before your diagnosis? What is the perception of the local people about breast cancer? Tell me how you felt after the surgery. What do you think of yourself now? What is your motivation to go on? Tell me about your support networks?

Memos were recorded immediately following the in‐depth interviews by the researchers, noting every participant's body language, facial expressions and responses observed during the interview. The study also documented perceptions and feelings as observed during the interview for every participant, as well as preliminary thoughts on emerging themes and theoretical assumptions [19]. For instance, Box 1 gives an account of the encounter with one of the participants.

BOX 1 **A memo written after interviewing participant Z.**

This was quite an eye‐opening interview for me because the interview process itself was odd, she was very determined throughout and kept asking for reassurance that she would neither be identified nor filmed. According to her accounts, she desired an interview during a clinic day, when the hospital's daily activities would naturally flow around her. She had told people that she was visiting relatives in the hospital, so no one would know that she was sick if they saw her. Her answers were usually monosyllabic and the interview was the shortest. The initial feeling was that the place of the interview was a hindrance because we kept getting interrupted, but she would not have it in any other place.

However, the interview was generally good. The revelation was that she was so paranoid about people finding out that she had breast cancer. So, the question is, why was she more concerned about her physical appearance to others than the experience of the mortal illness itself? Was she not aware that as treatment proceeded, the physical toll would begin to take effect, and become more visible and she may need some financial and emotional support for other treatments like surgery? Was this how more women in the course of the study will also pay as much attention to making sure that they are not identified with breast cancer? What is the motivation behind their desire to avoid identification?

The overall impression was that she was in denial and was not willing to accept that she had breast cancer because of her religious beliefs. To do so would mean acknowledging that she had a deadly, life‐changing disease. This perception was later evident in her narrative because even though she knew something was wrong with her breast. Initially, she did not go to the hospital because she did not want to find out if something ‘bad’ was wrong with her breast. This creates a new focus on the role of faith healing in treatment. Is denial a strategy in faith healing? How does faith‐based healing work? What is the role of spirituality in the breast cancer trajectory?

Case‐based memos were developed immediately after each interview. This allowed the study to capture initial ideas and compare participants' accounts and reflections. The comparisons enriched data analysis and guided further data collection for quality improvement. The quality of this study was further enhanced by ensuring that all interviews were digitally recorded, professionally transcribed in detail and the transcripts checked against the recordings. The study relied on purposive sampling for the initial small sample. The interview transcripts (from the initial small sample) were analysed immediately after each interview to justify theoretical sampling which increased the sample size. The immediate review of the interview created the opportunity to contact participants after each interview for conceptual reflections and clarification.

During data analysis, explanatory notes were developed, which enhanced the development of models relating abstract concepts to the social process. Further, in line with the grounded theory process specifically [19], data analysis commenced as soon as transcription began and was continuous as long as the need for new data was identified and until saturation was achieved. During this process, the data were coded and categorised. Memos/field notes were also used for comparisons until data saturation was achieved. The fundamental method for analysis with grounded theory was ensured, including openness throughout the study process. During the analysis, substantive and theoretical coding and constant comparison were also used to explain the disparities in the data. The codes were eventually combined into more abstract categories with properties to form the emerging framework. The first process was the coding and category development process, whereby line‐by‐line in vivo codes were developed from initial data, and then focused coding was conducted with colour codes previously agreed upon by the research team based on emerging concepts. Throughout the process, the researchers were engaged in triangulation of methods by reviewing the case notes of the participants, the focused group discussions (FGD) conducted with the ontology team and the researchers' memos. Also, theoretical sampling was used to test and elaborate on the categories until saturation was reached.

For context, data collection was obtained within 2 years; all the women in this sample had a mastectomy; some had already undergone surgery before the recruitment commenced, while others had surgery during the study. In the GTA, saturation must be achieved. It is the process in which all the concepts in the framework being developed have properties that describe these concepts and are well‐understood and (adequately) substantiated from the data. At the interpretation and analysis stage, this model/theory is expressed and presented as cohesive, abstracted, schematising explanations for understating the studied phenomena. Therefore, some of the participants were interviewed twice and some thrice until no new theme emerged and saturation was reached. The initial interviews lasted between 40 and 102 min because the interviewees were emotional. Subsequent interviews lasted between 30 and 48 min.

All participants chose their place of interview; some chose a place of worship, some wanted to meet at a friend or relative's house, some in the area where they were receiving traditional/alternative treatments and some in their homes. The hospital was the preferred interview location for only two participants.

## 3 Results

This study delves into women's illustrative narratives that specify the basis for explaining emergent self‐identities through the breast cancer journey. The framework or model presents the behavioural changes in agency that the women experienced during the three phases: prior to breast cancer diagnosis, treatment and post‐treatment. The model indicates how the self‐identities change in three phases: the initial pre‐diagnosis phase, transitional during treatment and post‐treatment/survivor phase. To inductively derive a typology, the self‐identities of the women living with breast cancer and their meaningful relationships were analysed. These relationships form the basis for type constructions and their properties, as presented in Table 2.

**TABLE 2:** Meaningful relationships and the development of type construction.

Type I and its properties	Type II and its properties		Type III and its properties
Valued self‐identity	Dependent self‐identity	Self‐determined identity	Diminished/devalued self
Every woman living with breast cancer (WLBC) regardless of time of life, religion and socio‐economic status. Social support is perceived to be high. Religiosity is perceived as high.	Young age of life. Social support is low. High belief in spiritual healing and alternative medicine. Tends to reject biomedical treatments.	Middle‐aged WBLC. Social support is high or not wanted. Accepts both biomedical and spiritual treatments.	Loss of self‐esteem and worth. Loss of sexuality and appeal. Does not want to be identified as a breast cancer survivor.

**FIGURE 1:**
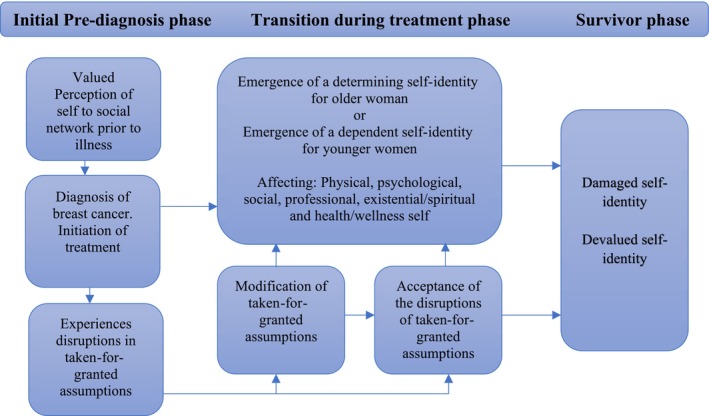
Typologies of self‐identity of women with breast cancer.

In these properties, which were inductively derived as contributing to the dominant themes in the women's narratives, exploring the lived experience of having breast cancer showed varied and unique perceptions. Although each participant observed and interpreted what was perceived uniquely, certain commonalities in the perception of agency emerged. These commonalities were more visible in the initial and final phases of their trajectories. However, the traumatic event of experiencing breast cancer was not a one‐off event but rather a continuum of events resulting from the rapid course that involved interactions with self and others in the diagnosis. It also involved the search for treatment, the treatment itself and post‐treatment for the patients. Figure 1 presents the eventual relation and development of the typologies of self‐identity of women with breast cancer.

### 3.1 Initial Phase: Valued Self‐Identity

In the first phase, every woman's insight of self was a valued one before diagnosis. The valued self‐identity was usually related to self‐perception—as beautiful and able to attract new partners—especially sexual and generally having meaningful relationships. The initial phase is that of a valued ‘whole’ woman (i.e., complete physical self), with the ability to function and fulfil roles as sexual partners, mothers, wives, friends and other social roles (i.e., social self). This, however, with a breast cancer diagnosis, self was re‐evaluated following the disruptions of everyday taken‐for‐granted assumptions about social relationships. Here, agency was passive for the very young woman, usually subordinated and dependent on significant and social others. One woman said

If I were told in my dreams that my people would run away from me because I have breast cancer, I would not have believed it. These are my family members and close friends! They started telling me about their problems and why they could not help me.

### 3.2 Transition Phase: Dependent Self‐Identity and Self‐Determined Identity

The transition phase is where the divide occurs, where the women's self‐identities begin to emerge and differentiate. The women emerged into two distinct self‐identities. This division became evident during diagnoses and treatment, characterised by the modification or acceptance of the disruptions of the taken‐for‐granted assumptions and the development of different self‐identity based on the woman's age and other socio‐cultural and economic factors.

#### 3.2.1 Type 1: Dependent Self‐Identity

This is the consciousness of self for the young married and unmarried woman with breast cancer. At this stage of their lives, young women are often economically and emotionally dependent on others. Women's roles are changing because of the cost and trauma of treatment (chemotherapy and mastectomy). The young woman is significantly less sexually attractive or appealing to her partner, cannot afford the cost of treatment and may be unable to attract new affection. The married young woman will have fertility issues, cannot nurture her child etc. This group of women experiences a shift in their self‐worth, which they perceive as diminishing in comparison to men. Because agency in this category is highly dependent on significant others, the self is passive, with most young WLBC unable to interpret, evaluate and map out their actions. Hence, the self is prone to manipulation and control. One of the women narrated as follows:

I had to undergo many procedures that they [my family] asked me to do because I needed help. For instance, my family forcefully took me to a woman [a traditional healer], who said that I should bring many things like a white goat, white chicken and white frog before she would do anything. She noted that it was a witch that had taken my breast. I refused. Then my people started mocking me, saying that if I refused to do what they [my family] wanted, they would leave me to die since that was what I wanted.

#### 3.2.2 Type II: Self‐Determined Identity

This is the other side of the coin for self‐identity during treatment. This is the consciousness of self for the more mature woman with breast cancer. Though roles and worth are changing in terms of sexual relations, fertility and menopausal issues, the agency is not passive. This category of women is not as emotionally and economically dependent as the other group. Here, there is the power to evaluate, negotiate and operate self‐reliantly because their agency is not dependent on others. One woman explained:

I was determined to find the money for treatment because I was afraid that if anything happened, who would take care of my children? I have to get well for the sake of my children. Ah, since they [doctors] told me it is cancer, I have had sleepless nights, and my children will suffer. So, I have to find the money. I sold some properties to cover treatment costs.

### 3.3 Final Phase: Devalued Self‐Identity

The psychosocial processes involved in confronting breast cancer are more intense in the final phase for all women, regardless of age and agency in the transition phase. This is the consciousness of self for all the women in this study after treatment and during the psychosocial processes (the new psychological self) involved in confronting breast cancer. In this phase, every respondent self‐identified as being diminished and degraded as a woman, that is, less feminine in body and roles, as their new normal. Even though some of their roles and activities, like motherhood, occupational and everyday activities, have returned, self is and remains devalued and becomes the new normality as they self‐identify as damaged because of the disfigurement of their bodies as a result of the loss of a breast. The reality of life manifests in an ‘incomplete’ body without the ‘femininity’ that comes with being a woman, including the deprivation of affective or sexual relationships. The devalued self‐identity is more pronounced for the young and unmarried because it diminishes their chances of attracting new affection or marriage. All the women perceived their selves as diminished, devalued or damaged. For example, two of the women described themselves as follows:

I have never had a serious relationship because of this mastectomy. I cannot look at myself in the mirror. Even after all these years, the scar is still ugly. I have not allowed even my adolescent daughters to see me naked, not talk of a man. Only my doctor has seen my breast since I had breast cancer…I feel incomplete and damaged. I do not want anyone to see my body.

I feel I am damaged. I am no longer a woman. My husband also does not touch me [sexually] anymore. I understand that this is due to my physical disfigurement. It has been 2 years and he has not looked at me [i.e., no sexual intercourse]. I know he can't stay 3 months without sex. I am no longer of value to him as a wife.

## 4 Discussion

Breast cancer in women under 35 is more than twice as prevalent in Black women than in other races [5, 27, 28]. Unfortunately, the prognosis is also poorer among (racial) minority groups with limited access to specialised healthcare [27]. The same story for women in developing countries where such specialised healthcare is a luxury. However, where treatment is available, it comes with social implications. In general, women often have to undergo treatments that alter their femininity in the childbearing age stage of life; they experience peculiar psychosocial issues, unlike the older women, regarding agency in their breast cancer trajectory. Most literature concerning breast cancer in the Global North often likens self or agency identity to survivor status [29]. In this study, women living with breast cancer think more of the damage already done to their femininity instead of survivorship. However, survivorship is an often‐contested multidimensional concept in culture and cancer discourse [10]. It started in the United States as a motivating psychosocial term to encourage people to learn to fight cancer [30].

The ‘new normal’ has emerged as a concept in illness experience analysis, particularly with young women with breast cancer and everyday life [31, 32, 33]. The concept explores the way and manner in which women rebuild their identities with the prospects of living longer lives with improved access to early screening and detection and newer treatment technologies in the reconstruction of everyday lives with breast cancer [34, 35, 36]. There are many studies on how people deal with their diseases or illnesses [36, 37, 38]. Trusson et al. [36] explored the new normal for women living beyond breast cancer treatment, especially after the end of treatment. Like Trusson et al., the participants in this study were somewhat afraid of the reality of an emerged new normal of an altered body and identity of life after mastectomy. Unlike Trusson et al.'s participants, the lack of finances, lack of access to improved technologies and treatments for breast cancer and complete mastectomy without breast reconstruction were new realities.

An essential reality of a history of breast cancer, especially as a young woman, is the impact on fertility and premature menopause. However, the added reality for Nigerian women is the heightened awareness of the embodied reminder of their bodies because of the total loss of interest in sexual intimacies by their partners, who reinforce these inadequacies by engaging in sexual relations with other women and sometimes marrying other women. This brings to the fore the influence of social structure and culture in a space where patriarchy is rife and the core values of a woman are to reproduce and nurture children.

This article focuses on Nigerian women's self‐identities after breast cancer treatment. The data provided by these women were the basis for developing a framework of self‐identity describing the process by which Nigerian women experience agency while living with breast cancer (Figure 1). The construction of a framework explored how illness identity is constructed through interaction with self and others in psychological and socio‐cultural contexts that influence the illness trajectory of having breast cancer and fill the gaps in literature from the Global South perspective.

Illness identity is a fascinating concept in chronic illness literature [39, 40]. Illness identity is the extent to which a chronic health condition is incorporated into someone's identity [41]. It expresses how much the disease has affected and dominated (social) self‐worth [41]. Other studies have explored illness identities [39, 40, 41, 42, 43], with resulting frameworks of what illness identity means to the study population bearing that the models reflected different views from different chronic illnesses. This article's overarching framework of illness identity comprises three constructs: valued self, determined/dependent self and devalued self‐identities (Table 2).

This study discovered that the social context of a Nigerian woman with breast cancer necessitates situating an understanding of meaning within the context and timing of illness as it affects self‐identity ([44]). Breast cancer affects the various dimensions of self including the physical self (bodily changes, disfigurement and physical limitations), the psychological self (emotional impact, coping mechanisms and resilience), the social self (changes in social roles, support networks and stigma), the professional self (impacts on work or career, economic consequences), the existential or spiritual self (grappling with morbidity and finding meaning), health and wellness self (adopting healthy lifestyles, self‐care practices). Self‐identity is the different ways in which people choose to define themselves, and could reflect how the disease process affects them. The women developed their self‐identities throughout their illness journey and in line with the time of life (age) of their lives. In the first phase, the women had a heightened sense of worth to themselves, their bodies, family and social networks. However, breast cancer diagnosis shakes these assumptions about possessing a smoothly functioning body [18]. It further disrupts previously taken‐for‐granted assumptions about relations with family and friends, especially when social support is limited.

In Phase II, the development and refinement of the women's self‐identities became more apparent. The self‐identity of the younger woman with breast cancer was a dependent one. In this sense, she was more likely to be financially/economically dependent on her significant others. This economic vulnerability significantly affects their illness behaviour because the cost of treatment and follow‐up care can place an enormous burden on the patient, their family and others' support. In this category, as the young woman with breast cancer grappled with her illness and treatment, her family members grappled with financial concerns. Consequently, they encouraged her to explore alternative care methods that were more affordable, such as faith healing and traditional medicine. The use of traditional medicine is significantly high in Africa [45] and for breast cancer [46, 47]. On the other hand, the older woman had a determined self‐identity, where she was less dependent economically and emotionally. She is more established in her job, has access to economic properties to sell or rely on her support networks, and is more financially secure than the younger woman, so support is more readily available.

The third phase presents a disruption of the sense of wholeness of body and self and gives a damaged and devalued self‐consciousness. This is related to Bury's biographical disruption [18, 48]. This phase clearly shows that a woman with breast cancer has an increased self‐concern compared with her initial valued self‐image. That heightened self‐concern raises several dilemmas because they now see themselves and their lives as damaged and devalued. This finding is also consistent with McCann et al.'s [49] longitudinal study about identity transition: moving between health and illness and transitioning to the future. McCann et al. [15] observed that identity transition emerged due to the changes and adaptations to their diagnosis. Rees [39] argued that the uncertainty and liminality of living with breast cancer affect the identity of young women, especially regarding fertility and menopause. However, some women accept the certainty that there is no returning to the normal state prior to cancer and recreating a new normal. The young women's perceptions about the future were altered, with fears about recurrence and their maternal roles in the future in jeopardy. This article also showed that examining cancer experiences within the first year following diagnosis could provide a significant understanding of the impact of cancer on one's identity from the moment of diagnosis [39].

In using her version of disruptions and self‐identities, that is, how people repair the loss of self due to a chronic illness, Charmaz [19] explained how people modify their identities to include ignoring, minimising, struggling, reconciling and embracing. For Charmaz, most people choose the adaptation identity because adaptation aids acceptance. However, the most pertinent experience comes with the ‘struggling identity’ to living without the breast and the altered self vis a vis the sense of self before mastectomy. The scar was a constant reminder of the mutilated body, the sense of loss of an ideal body and the reality of lack of sexual intimacy. While gender inequalities are common in most societies, the gender gap in Africa is quite wide, with equally high unequal power relations that make women vulnerable in almost all relations with men [50, 51]. For instance, cultural and patriarchal norms enable husbands of these women with breast cancer to dislodge or replace them easily because of the idealised body. This also reinforces the struggle with identity impairment because bodily appearance alters social and self‐identities.

Within this process of developing a new self‐identity, the women in this study tended to glide, as Morse [52] explained, up and down the hierarchy of identities with the formation of self, which occurs through social interactions. Charmaz [18] also demonstrated that people go through three stages before arriving at ‘adaptation self‐identity to the altered self’. A bit of Charmaz's identity hierarchy was also visible in women. In contrast, a few of the women desired to retain the former ‘restored self’ (especially for those in the self‐determined identity), and there was no desire by any of the women to have a ‘supernormal self‐identity’. The contingency hierarchy was more overriding in the narratives of the women, especially for those who perceived the source of cancer as a spiritual attack. The salvaged self‐hierarchy is, however, what aptly describes the identities of most of the women. However, the few diagnosed early and who were in the post‐treatment stage assumed the ‘adaptation self‐identity to the altered self’ silently or secretly because they ultimately did not want to be identified as survivors.

Like Charmaz [17] and Morse [52], the self‐identities that emerged from the illness experience in this study were dependent upon the type and degrees of illness, the time of life, the meaning of the illness experience and the individual's expectation of and for the self as well as interactions with others in the social world. They scrutinise their encounters with others for hints of discreditation and negative reflections of self. Hence, they become particularly perceptive of others' negative reflections as a rejection of the new self. The identity goal is to survive for the sake of life but not live and function fully as a sexual woman.

Even when the visible signs of the scar were fading for most of the participants, there is a constant reminder of the devalued self‐identity. Hence, breast cancer lived‐experience is often hidden and implicit in Nigeria, because appearance affects women more than men, especially in the African space where gender inequalities inevitably make women heavily dependent on men for survival and where female autonomy is often minimal or compromised. With the diagnosis and limited choice of treatment for breast cancer, a woman is glaringly reminded that her essence is directly related to the ideal body where the breast is a most necessary part of being a woman. The women are thus in a perpetual flux of the psychosocial processes involved in confronting breast cancer.

## 5 Conclusion

This study has made an essential contribution to the knowledge base on the development of breast cancer self‐identities among Nigerian women. This is because most studies of self‐identities in chronic illness are from the Global North. The findings showed that women with breast cancer of different ages share common concerns and anxieties that disrupt taken‐for‐granted assumptions regarding agency before diagnosis, treatment and post‐treatment phases. To understand the significance of these disruptions for women, reconnoitring the consciousness of self‐identity before and after the disease diagnosis is necessary to comprehend the meaning of living with breast cancer. For survivors, having breast cancer translates to a significant sense of vulnerability, primarily because breast cancer significantly alters womanly essence.

Further studies can use the knowledge of the context to further the explanation of the typologies and for possible theory development. These findings can also inform the development of psychosocial and other supportive interventions. Furthermore, understanding health and illness behaviour about breast cancer may help healthcare providers to understand illness trajectories and the profound psycho‐emotional impact of breast cancer.

## Author Contributions

**Aisha Abimbola Adaranijo:** conceptualization (lead), data curation (lead), formal analysis (lead), funding acquisition (equal), investigation (lead), methodology (lead), project administration (lead), resources (lead), software (lead), validation (lead), visualization (lead), writing – original draft (lead), writing – review and editing (supporting). **Jimoh Amzat:** conceptualization (supporting), formal analysis (supporting), funding acquisition (supporting), investigation (supporting), methodology (supporting), project administration (supporting), resources (supporting), supervision (lead), validation (supporting), visualization (supporting), writing – original draft (supporting), writing – review and editing (lead). **Dejo Abdulrahman:** conceptualization (supporting), formal analysis (supporting), investigation (supporting), methodology (supporting), project administration (supporting), supervision (supporting), validation (supporting), visualization (supporting), writing – original draft (supporting), writing – review and editing (lead). **Kehinde Kazeem Kanmodi:** funding acquisition (supporting), resources (supporting), writing – review and editing (supporting).

## Ethics Statement

The study was approved by the Institutional Review Board of the Federal Medical Centre Lokoja, Kogi State, Nigeria (reference number: FMCL/MED/115/II/212). Written informed consents were obtained from all participants, and all participation was strictly voluntary and confidential. This study did not exceed the scope of the original ethics committee approvals.

## Consent

Written informed consents were obtained from all participants.

## Conflicts of Interest

The authors declare no conflicts of interest.

## Data Availability

The data that support the findings of this study are available from the corresponding author upon reasonable request.
